# Rhizosphere assisted biodegradation of benzo(a)pyrene by cadmium resistant plant-probiotic *Serratia marcescens* S2I7, and its genomic traits

**DOI:** 10.1038/s41598-020-62285-4

**Published:** 2020-03-24

**Authors:** Rhitu Kotoky, Piyush Pandey

**Affiliations:** Department of Microbiology, Assam University, Silchar, Assam 788011 India

**Keywords:** Applied microbiology, Environmental microbiology

## Abstract

*Melia azedarach-*rhizosphere mediated degradation of benzo(a)pyrene (BaP), in the presence of cadmium (Cd) was studied, using efficient rhizobacterial isolate. *Serratia marcescens* S2I7, isolated from the petroleum-contaminated site, was able to tolerate up to 3.25 mM Cd. In the presence of Cd, the isolate S2I7 exhibited an increase in the activity of stress-responsive enzyme, glutathione-S-transferase. Gas Chromatography-Mass spectroscopy analysis revealed up to 59% *in -vitro* degradation of BaP after 21 days, while in the presence of Cd, the degradation was decreased by 14%. The bacterial isolate showed excellent plant growth-promoting attributes and could enhance the growth of host plant in Cd contaminated soil. The 52,41,555 bp genome of isolate *S. marcescens* S2I7 was sequenced, assembled and annotated into 4694 genes. Among these, 89 genes were identified for the metabolism of aromatic compounds and 172 genes for metal resistance, including the efflux pump system. A 2 MB segment of the genome was identified to contain operons for protocatechuate degradation, catechol degradation, benzoate degradation, and an IclR type regulatory protein *pcaR*, reported to be involved in the regulation of protocatechuate degradation. A pot trial was performed to validate the ability of S2I7 for rhizodegradation of BaP when applied through *Melia azedarach* rhizosphere. The rhizodegradation of BaP was significantly higher when augmented with S2I7 (85%) than degradation in bulk soil (68%), but decreased in the presence of Cd (71%).

## Introduction

The co-contamination of polycyclic aromatic hydrocarbons (PAHs) and metals like cadmium occur in various ecosystems and are priority pollutants due to their potential toxicity, mutagenicity, and carcinogenicity. According to Lin *et al*. (2006), the co-contamination of hazardous wastes with organic and metals like cadmium pose negative health effects to human and ecosystem^1^. Microbial processes for removal of pollutants provide a promising, and cost-effective approach for cleanup of PAH and metal-polluted environments. However, the co-contamination of other inorganic xenobiotic compounds complicates the biodegradation of organic compounds due to their harmful effects on the biological system. Still, the bacterial species, which harbor functional genes for both the degradation of a range of different hydrocarbon compounds and resistance to metal toxicity^2–4^ presents a promising biotechnological advantage for clean-up of such environments.

The technology of microbe assisted phytoremediation has become a promising method for the removal of petroleum contaminants from polluted soils that include procedures like phytoaccumulation, phytovolatilization, phytoextraction, rhizodegradation^5^. Currently, rhizoremediation or rhizodegradation technique is more recognized which involves both, plants and their associated rhizospheric microbes for degradation/removal of contaminants. The plant-microbe association may occur naturally or get established by introducing specific efficient microbes^6^. However, the application of these bioremediation methods is limited because it takes more time and affected by several other factors like contaminant distribution, soil nutritional status, soil water pH, temperature and oxygen supply affect the process of degradation^7^. Therefore, in the present study bacterial isolates with multiple characteristics had been isolated and evaluated for their prospects for rhizosphere mediated bioremediation of contaminants.

In this study, several isolates were screened for unique abilities to degrade benzo(a)pyrene (BaP), in addition to resistance for Cd and have plant growth promotion (PGP) attributes too. One of the isolates, *Serratia marcescens* S2I7, isolated from petroleum-contaminated soil was found to be highly resistant to a higher concentration of cadmium and have PGP traits, and also could degrade BaP efficiently. However, no previous study has reported any *Serratia marcescens* strain that degrades PAHs with the potential to be used for bioremediation.

Recent advances in next-generation sequencing technologies have led to an increase in the sequencing of complete genomes of beneficial organisms and to characterize their genetic potential. Several studies have reported complete or draft genome sequence and characteristics of bacteria belonging to genus *Serratia*, a Gram-negative bacterial genus of Gammaproteobacteria family^8,9^. To date (January 2020), there are more than 540 genome assemblies and annotation reports of *S. marcescens* strains present in the database of NCBI (www.ncbi.nlm.nih.gov) reported from different environmental and clinical samples. However, the strains from clinical and environmental samples could be placed in a completely different clade when analyzed through multi-locus sequence typing (MLSTs), which suggests a clear association between the source and genotype of an organism^10^. Considering these, the present work was undertaken to study the genome of *S. marcescen*s S2I7, a Cd-tolerant bacterium with PGP properties that can efficiently degrade PAHs. To evaluate the potential of this isolate for bioremediation of hydrocarbon contamination sites, its genome was studied, along with analytical analysis of PAH degradation *in-vitro* conditions and also with the association of *Melia azedarach* plant.

## Results

### Isolation of BaP degrading bacteria, resistant to Cd

A library of bacterial isolates was maintained, isolated from contaminated soil. Among several bacterial isolates, 14 bacterial strains were found to be resistant for cadmium and were also able to utilize BaP efficiently as the sole source of carbon. The isolates S2I7, S2I3, and S2I5 showed maximum tolerable concentration for Cd (3.25 mM, 2.75 mM, and 3.0 mM respectively) whereas the strain S1I2 showed the lowest resistance to Cd (0.5 mM) (Supplementary File 1). The isolates were primarily screened for BaP degradation with a colorimetric assay. The isolates S1I26, SR1, S2I5, S1I7, S1I5, S1I8, S2I7, PDB4 showed more than 45% of degradation. Whereas strain S1I5 showed the highest (66%) degradation, followed by isolate S1I8 (60%), while the isolate S1I2 showed the lowest degradation (9%) of BaP in a liquid medium. The results are given in Supplementary File 2.

### Plant growth-promoting attributes of the isolate

The isolates were analyzed for their PGP attributes. Isolates S2I7, S1I26, S1I1, SR1, S1I8, and S1I7 were found promising for PGP attributes. Among these isolates, S2I7 was found to be best as it could solubilize inorganic phosphate in Pikovaskay’s agar medium and showed significant production of IAA, (49.8 micrograms per milliliter after 144 h of incubation) too. Moreover, the isolate was also found to be positive for the production of siderophore when checked on CAS medium, as well as showed moderate production of HCN (Supplementary File 3).

### Characteristics of the selected isolate

The 14 bacterial isolates capable to degrade BaP, were further screened to select the most efficient bacterial isolate, based on its Cd-resistance and PGP attributes. Venn analysis was prepared to keep the threshold value of Cd resistance at 2.75 mM whereas IAA production value at 20 µg/ml (Supplementary File 4) among these 14 isolates. Therefore, based on initial experiments and screening processes, the strain S2I7 was selected for further studies. Molecular phylogeny analysis revealed the strain S2I7 belong to genus *Serratia*, (class- gammaproteobacteria) and closely related to *Serratia marcescens* strains. The isolate *S. marcescens* S2I7 has been submitted to Microbial Culture collection center, National center for microbial resource, Pune (NCMR) with the accession no MCC-3537 under the public domain section.

**Figure 1:**
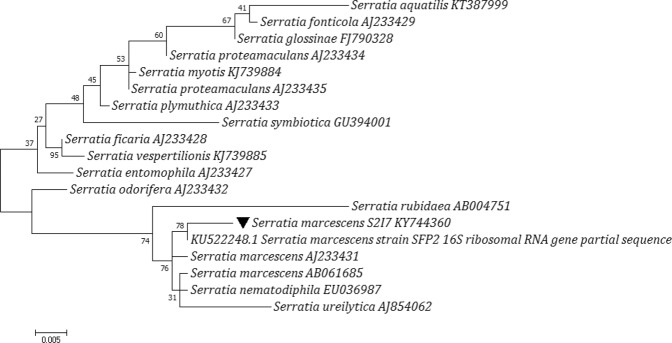
Molecular Phylogenetic analysis of *S. marcescens* S2I7 by Maximum Likelihood method^53^. The tree is drawn to scale, with branch lengths measured in the number of substitutions per site. The analysis involved 19 nucleotide sequences. All positions containing gaps and missing data were eliminated. Evolutionary analyses were conducted in MEGA75^54^.

*S. marcescens* S2I7 was Gram-negative, motile, rod-shaped facultative aerobic bacteria that produced red color pigment (prodigiosin) when grown at a temperature below 30 °C. Prodigiosin is commonly produced by environmental isolates of *S. marcescens*, but not the clinical isolates^11^. The phylogenetic analysis of the 16S rDNA sequences of the selected strains in the present study showed two major clades among various *Serratia* species. The strain S2I7 was found to be grouped in the clade of *S. marcescens* where it was closely related to strain *S. marcescens* SFP2 (KU522248). Other species of the genus were found to be grouped in another clade (Fig. 1).

### Catechol dioxygenase enzyme activity of *S. marcescens* S2I7

The isolate *S. marcescens* S2I7 showed the presence of both enzymes, i.e. C12D and C23D, in the intracellular extract of bacteria, when grown with BaP as the sole source of carbon. The C12D activity decreased with the duration of incubation and it was highest (14 U/ml) after the initial 2 days of incubation, whereas it was lowest after 5 days (3 U/ml). The activity of C23D increased with the duration of incubation and the highest activity was noted after 5 days of incubation (Supplementary File 5).

### Quantitative estimation of PAH degradation in the presence of Cd and/or succinate as another carbon source

**Figure 2:**
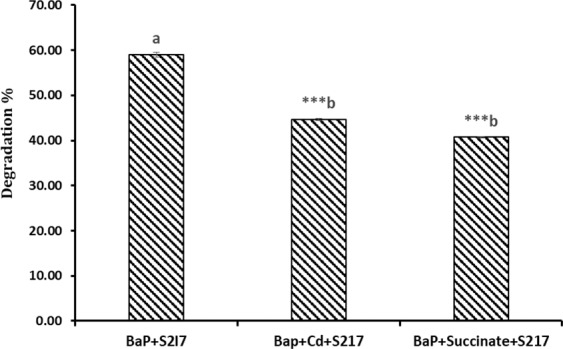
*In-situ* Degradation percentage of BaP inoculated with *S. marcescens* S2I7 and effects of Cd and succinate on it. One-way ANOVA was done followed by post-hoc analysis, where groups bearing the different superscript are significantly different from each other. Values are significantly different from control: *p < 0.05; **p < 0.01; ***p < 0.001.

Quantitative degradation of BaP was analyzed by GC-MS analysis and the effects of Cd and succinate were studied. Analysis for spectrogram showed the peak of BaP (m/z-252, retention time 33.94 in Supplementary File 6) and the concentration was analyzed based on the area of the peaks. GC-MS analysis showed that the degradation of BaP was 59% after 21 days. In the presence of Cd, the BaP degradation was found to decrease by 15%, and in the presence of succinate, it decreased by 18% (Fig. 2). The mass spectrometer analysis revealed different metabolites such as phthalate, salicylic acid, 1,2-benzene dicarboxylic acid, butyl 2-ethylhexe, 1-butyl 2-cyclohexyl phthalate, that were formed during the degradation of BaP.

### Genomic insights for *S. marcescens* S2I7

**Figure 3:**
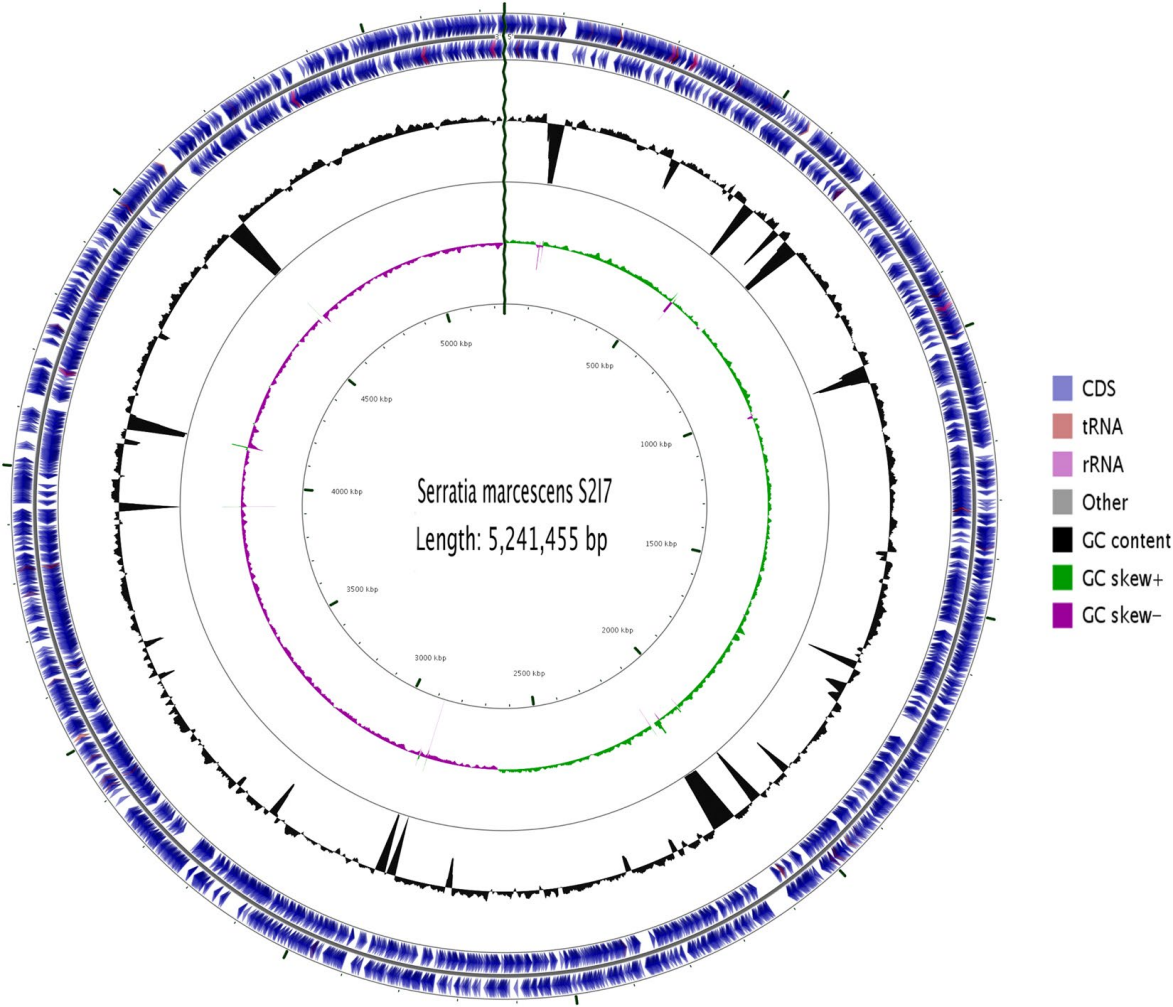
Circular map of the genome of *S. marcescens* S2I7. From outside to the center, genes on the forward strand, genes on the reverse strand, RNA genes, GC content, and GC skew.

The genome of *S. marcescens* S2I7 consists of one circular chromosome of 5241555 bp with 60.1% GC content (Fig. 3). The genome of *S. marcescens* S2I7 includes 4,533 protein-coding genes, 81 tRNA genes, and 22 rRNA genes and a total of 47 pseudogenes. The predicted coding sequences were translated, compared and searched against the NCBI protein non-redundant database, the Clusters of Orthologous Groups (COG) and KEGG databases. The whole-genome project is deposited in GeneBank under the accession number CP021984 and currently available online at NCBI/Genebank database.

#### Identification of genes responsible for PAH degradation

**Figure 4:**
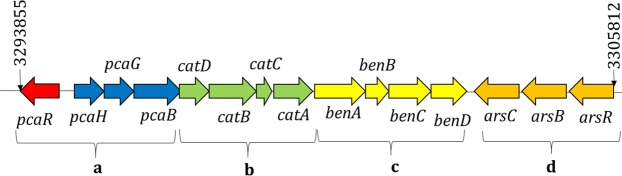
Dioxygenase gene clusters for a breakdown of aromatic compounds and arsenic resistant along with their respective position in the genome of *S. marcescens* S2I7. a-gene cluster for degradation of protocatechuate; b- gene cluster for degradation of catechol; c-gene cluster for degradation of benzoate and d-gene cluster for resistance to arsenic.

The genome of the strain contains 89 genes that were identified to be involved in the metabolism of aromatic compounds. One cluster contained several genes together for the degradation of aromatic compounds. This segment contains operon for protocatechuate degradation, catechol degradation, and benzoate degradation. Besides, in the downstream of this cluster, there also contains an operon for the arsenic efflux pump system (Fig. 4).

The other selected genes and operons are included in Table 1 along with their respective positions in the genome of *S. marcescens* S2I7. The genome contains more than one copy of some genes, where genes *pcaD, pcaF, pcaI, pcaJ* have been reported to be involved in more than one pathway of aromatic compound degradation.

**Table 1:** Selected aromatic compound catabolic genes with their respective position in the genome of *S. marcescens* S2I7 (Retrieved through RAST server^44^ and IMG database^12^).

Gene	Product	Pathway	Position
*benB*	benzoate 1,2-dioxygenase beta subunit (EC 1.14.12.10)	benzoate degrdation	3301335–3301832
*benA*	benzoate 1,2-dioxygenase alpha subunit (EC 1.14.12.10)	-do-	3299956–3301338
*benD*	1,2-dihydroxycyclohexa 3,5 diene 1 carboxylate dehydrogenase (EC 1.3.1.25)	-do-	3302876–3303658
*benK*	benzoate MFS transporter	-do-	391020–392348
*benE2*	benzoate transporter protein	-do-	2438103–2436916
*benC*	benzoate 1,2-dioxygenase	-do-	3301848–3302873
*bphC*	biphenyl 2,3 diol 1,2 dioxygenase (EC 1.13.11.39)	Biphenyl degradation	1276802–1277311
*bphJ2*	acetaldehyde dehydrogenase (EC 1.2.1.10)	-do-	2949116–2951788
*C23D*	catechol 2,3-dioxygenase (EC 1.13.11.2)	Meta cleavage pathway of Catechol degradation	659279–660091
*HMSD*	2-hydroxumuconic semialdehyde dehydrogenase	-do-	657729–659183
*catA*	catechol 1,2-dioxygenase (EC 1.13.11.1)	Ortho cleavage pathway of catechol degradation	3298937–3299866
*catB*	muconate cycloisomerase (EC 5.5.1.1)	-do-	3297430–3298545
*catC*	muconate isomerase (EC 5.3.3.4)	-do-	3298563–3298853
*catD/pcaD*	beta-ketoadipate enol-lactone hydrolase (Ec 3.1.1.24)	-do- Or Protocatechuate branch of beta-ketoadipate pathway	3296665–3297426, 1495835–1495026
*catF/pcaF*	beta-ketoadipyl CoA thiolase (EC 2.3.1.-)	-do-	3295538–3296656, 1963532–1962321
*catI/pcaI*	3-oxoadipate CoA-transferase subunit B (EC 2.8.3.6)	-do-	3294796–3295476
*catJ/pcaJ*	3-oxoadipate CoA-transferase subunit A (EC 2.8.3.6)	-do-	3294796–3295476
*HT*	hydroxybenzoate transporter	Hydroxybenzoate degradation	4372146–4373495, 890045–888705
*pcaR*	pca regulon regulatory protein	Protocatechuate branch of the beta-ketoadipate pathway	3293855–3293022
*pcaC*	4-carboxymuconolactone decarboxylase (EC 4.1.1.44)	-do-	1576629–1576961
*pcaI2*	succinyl-CoA:3-ketoacid-coenzyme A transferase subunit A (EC 2.8.3.5)	-do-	604665–605363
*pcaJ2*	succinyl-CoA:3-ketoacid-coenzyme A transferase subunit B (EC 2.8.3.5)	-do-	605375–606028
*SH*	salicylate hydroxylase (EC 1.14.13.1)	Salicylate & Gentisate degrdation	2508248–2507097, 1544654–1543419
*salD*	4-hydroxybenzoate transporter	-do-	4372146–4373495
*MI*	maleate cis-trans isomerase (EC 5.2.1.2)	-do-	2506290–2505538
*QuiB*	3-dehydroquinate dehydratase	Quinate degrdation	4732654–4733106
*iqoA*	isoquinoline 1-oxidoreductase alpha subunit (EC 1.3.99.16)	N-heterocyclic aromatic compound degradation	2510259–2510801, 2524817–2525275, 2700505–2700032
*iqoB*	isoquinoline 1-oxidoreductase beta subunit (EC 1.3.99.16)	-do-	2522595–2524817, 2525287–2526591, 2697748–2696450, 2700035–2697777

#### Identification of genes for resistance to metals

The strain *S. marcescens* S2I7 was found to be highly resistant to metal cadmium. The genomic characteristics of the strain has revealed the presence of several genes responsible for the resistance to arsenic, cadmium, cobalt, copper, nickel, zinc, etc. Cadmium metal efflux protein may be responsible for its resistance to it (Table 2).

**Table 2:** Genes responsible for the resistance to different metals present in the genome of *S. marcescens* S2I7 with their respective positions (annotated and retrieved from RAST analysis^44^ and IMG database^12^).

Gene	Product	Position
*czcD*	cobalt-zinc cadmium resistance protein	5143989–5143087
		1418025–1417165
		2719966–2718590
*cpx*	periplasmic zinc resistance associated protein	5144463–5144122
*cpxR*	copper sensing two-component response regulator	5144750–5145448
*cpxA*	copper sensory histidine kinase	5145445–5146839
*mgtE*	Mg/Co/Ni transporter	2721524–2720502
*czrR*	DNA binding metal response regulator	1621127–1621798
*TRcd*	Cd(II)/Pb(II) responsive transcriptional regulator	2484199–2484618
*CSA*	Cd/Zn/Copper/Silver efflux P-type ATPase	238321–236003
*CIA*	copper translocating P-type ATPase	1211807–1209096
*CRD*	copper resistance protein D	2091563–2092408
*copC*	copper resistance protein C precursor	2091141–2091524
*arsR*	arsenic resistance operon repressor	3305812–3305486
*arsB*	arsenic efflux pump protein	3305416–3304127
*arsC*	arsenate reductase	3304112–3303681
*scsA*	Putative copper binding protein	3746041–3745679
*scsB*	Suppressor for copper sensitivity	3745629–3743593
*scsC*	Suppressor for copper sensitivity	3743596–3742880
*scsD*	Suppressor for copper sensitivity	3742887–3742387
*cutF*	copper homeostasis protein	4187312–4186629
*cutE*	copper homeostasis protein	1309974–1308445
*corC*	magnesium and cobalt efflux protein	1310860–1309982

#### Identification of genes for plant growth-promoting attributes

**Figure 5:**
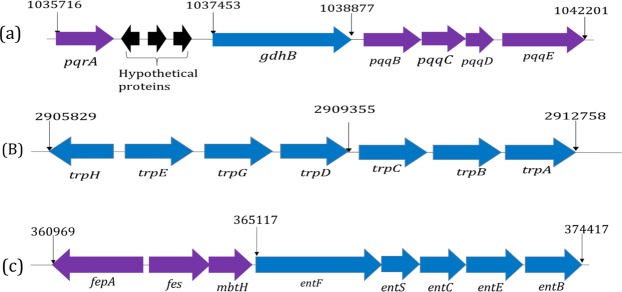
Gene cluster responsible for different plant growth promoting attributes present in the genome of *S. marcescens* S2I7, (**a**) gene cluster for phosphate solubilization; (**b**) structural gene cluster of tryptophan biosynthesis pathway (*trpHEGDCBA*); (**c**) structural gene cluster for biosynthesis of enterobactin siderophore.

The genomic sequence of *S. marcescens* S2I7 revealed the presence of a gene cluster for phosphate solubilization (Fig. 5(a)). The gene *gdhB* (1424 bp) contains four *PQQ* related genes in the downstream region, while in the upstream sequence contain three hypothetical proteins and the regulatory sequence *pqrA*. Moreover, the genome of *S. marcescens* S2I7 contains gene cluster *trpEGDCBA* that encode key enzymes in the tryptophan biosynthesis pathway (Fig. 5(b)) which is related to multiple biological processes, including IAA biosynthesis. Besides, the genome contains the key protein indole-3-pyruvate decarboxylase (*IPDC*) (1670 bp) that follows IPyA pathway for biosynthesis of IAA^9^. Genome characteristics of the strain showed that it produces ‘enterobactin’ type of siderophore that is encoded by a gene cluster (*entBECSFH*) present in the position 360969-374417 (Fig. 5(c)).

#### Identification of genes for stress response

Genes related to stress are important for the bacteria to survive under abiotic stress in the contaminated sites. Different genes for stress response were identified in the genome of *S. marcescens* S2I7 including enzymes associated with oxidative stress like catalase (*katA*, COG0753, position 3664475-3665935), peroxidase (*ycdB*, COG2837, 3167099-3165801) superoxide dismutase (*sodB*, COG0605, 76901-76236) and glutathione peroxidase (*gpo*, COG0386, position 2378493-2378008). Moreover, cold shock protein-encoding genes *cspB* (COG1278, position 1874241-1874462), *cspC* (3320090-3319878), *cspD* (1833609-1833493), *cspE* (1276647-1276156), were also detected in the genome along with different heat shock protein-encoding genes *hspQ* (COG3785, position 1931389-1931018), *hslJ* (COG3187, 2808804-2809232), *hslR* (COG1188, 4935707-4936114), *ibpA, ibpB* (COG0071, 43866-44279; 44369-44815).

**Figure 6:**
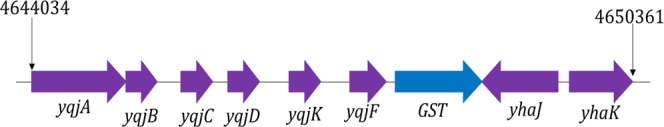
Conserved gene cluster of Enterobacteriaceae family containing glutathione s-transferase (GST) in the genome of *S. marcescens* S2I7.

The genome of *S. marcescens* S2I7 contains multiple GST genes belonging to the different superfamily of GSTs. Among these, the strain has a gene cluster, conserved to Enterobacteriaceae family containing the GST gene with multiple cellular membrane proteins (Fig. 6). The gene GST in the cluster has a transcriptional regulator (*YhaJ*) of the LysR family at its downstream in the opposite direction.

#### Comparative analysis of the genome

The genome of *S. marcescens* S2I7 was analyzed and compared with other 522 genomes of *S. marcescens* with the sequences available in the database of NCBI (retrieved on January 2020). The genomic tree was prepared according to the distance matrix, based on which S2I7 was found to be closely related to the strains *S. marcescens* EGD-HP20, EGD-HP20_1 and WW4 with the symmetric identity of 96.54%, 96.28%, and 96.12% respectively. The genome of *S. marcescens* S2I7 was found to be distantly related to the strains *S. marcescens* N2 and *S. marcescens* NCTC13920 with the symmetric identity of 56.56% and 74.07% respectively.

**Figure 7:**
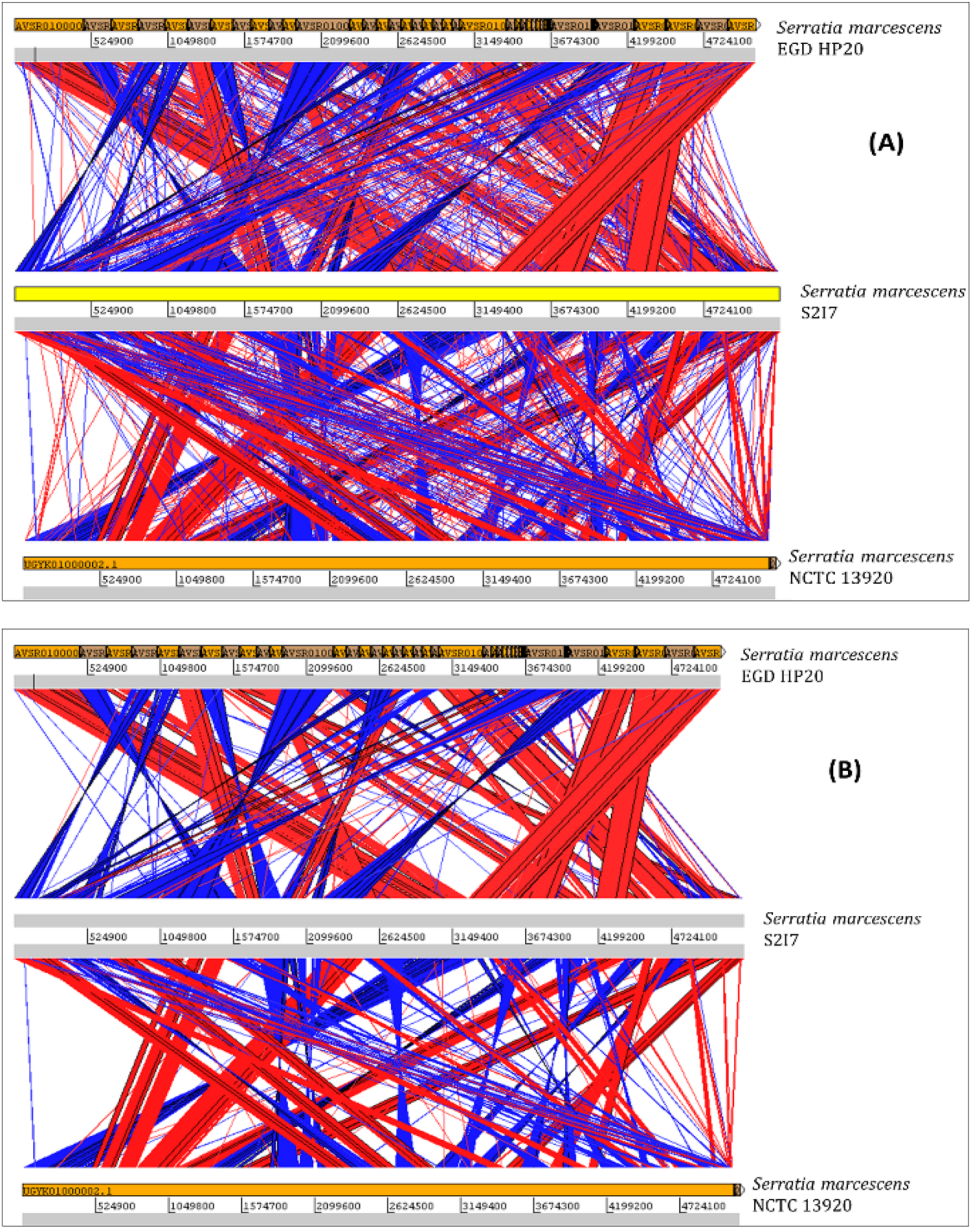
Artemis comparison analysis between genomes of *S. marcescens* S2I7 with its one closely related strain (EGDHP20) and one distantly related strain (NCTC13920). The analysis was done with 50% identical value and minimum cut off value of 28 (default, **A**) and minimum cut off value of 500 (**B**).

Then comparison analysis was done based on nucleotide sequences for S2I7 by Artemis Comparison Tool (ACT) with its most closely related strain (EGD-HP20) and one of the distantly related strain- NCTC13920, which was a clinical isolate. The result of genome alignment analysis based on the nucleotide sequence alignment between two genomes is given in Fig. 7.

**Figure 8:**
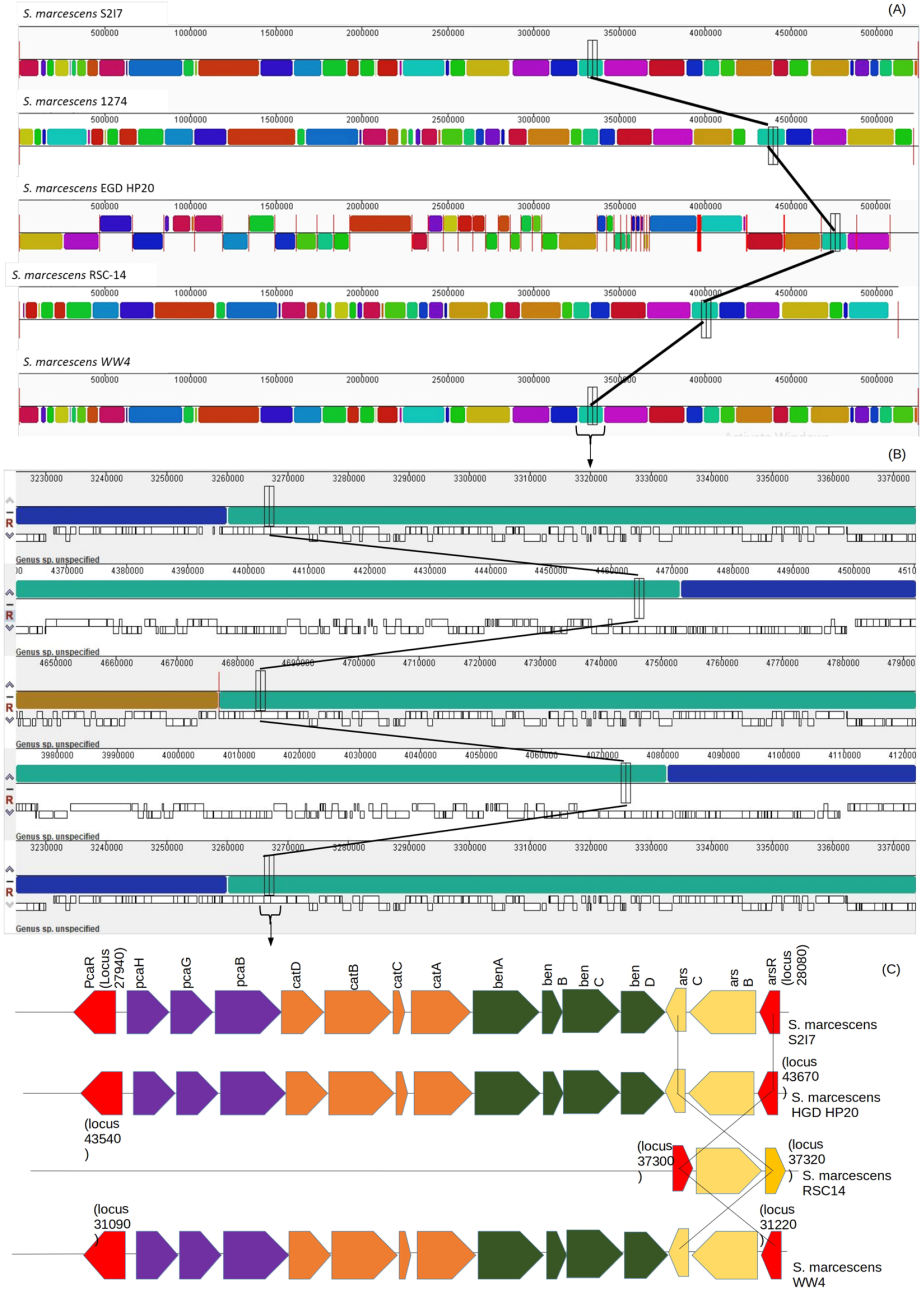
Comparative analysis of genetic features among five *Serratia* isolates. Arrangement of locally colinear blocks (LCBs) in the genomes of the isolates (**A**), enlarged one of the LCBs (**B**) and synteny analysis of a gene cluster containing catechol and benzoate degrading genes (**C**).

Apart from this, a comparative analysis of the genetic features of the genome S2I7 was done with four other *Serratia* genomes (Table 3) using Mauve software. Mauve can align orthologous and xenologous regions among two or more genome sequences where it identifies conserved segments in the genome rearrangements known as Locally Collinear Blocks (LCBs). Similar color blocks represent orthologous regions (Fig. 8(A)). We kept the genome sequence of S2I7 as query sequence and the sequences of other genomes were arranged relative to it. LCB appeared above or below the centerline which indicates forward or reverses the orientation of the sequences respectively relative to the query genome sequence.

**Table 3:** Genomic features of *S. marcescens* strains S2I7, 1274, RSC-14, EGD-HP20, WW4.

Organism	Strain	Bioproject	Accession No.	Size (Mb)	GC%	Gene No.	Protein	rRNA	tRNA	Pseudogene	Characteristics	References
*Serratia marcescens*	S2I7	PRJNA388843	CP021984	5.24	59.5	4694	4516	22	81	64	benzo(a)pyrene degrading soil bacteria	This study
*Serratia marcescens*	WW4	PRJNA886559	CO003959	5.2	59.5	4987	4827	22	81	46	Biofilm forming bacterium isolated from paper machine. Under condition of P limitations, exhibit intergenic inhibition of Pseudomonas	^55^
*Serratia marcescens*	RSC14	PRJNA294721	CP012639	5.12	59.6	4849	4673	22	87	65	PGP bacteria that alleviates cadmium stress in host plant	^9^
*Serratia marcescens*	1274	PRJNA371353	CP019927	5.21	59.8	4979	4285	18	82	582	Plant associated environmental isolate	^12^
*Serratia marcescens*	EGD HP20	PRJNA211617	AVSR00000000	5.08	59.8	4818	4728	5	79	6	Degrade poultry waste	^12^

In the zoomed image of the LCBs, we can see the annotated features of the genomes and their homology (Fig. 8(B)). From the annotated features of the LCB, we did synteny analysis or compared the arrangement of the gene cluster containing catechol, benzoate degradation, and arsenic resistance genes among the sequences (Fig. 8(C)). Synteny analysis of the segment showed the presence of an exact arrangement of the gene cluster in the genome of *S. marcescens* S2I7, *S. marcescens* EGD HP20, and *S. marcescens* WW4. However, in the genome of *S. marcescens* 1274, the cluster has been missing completely and in the genome of *S. marcescens* RSC-14, only the operon for arsenic resistance has been found in the opposite orientation.

#### Orthologous protein cluster analysis

**Figure 9:**
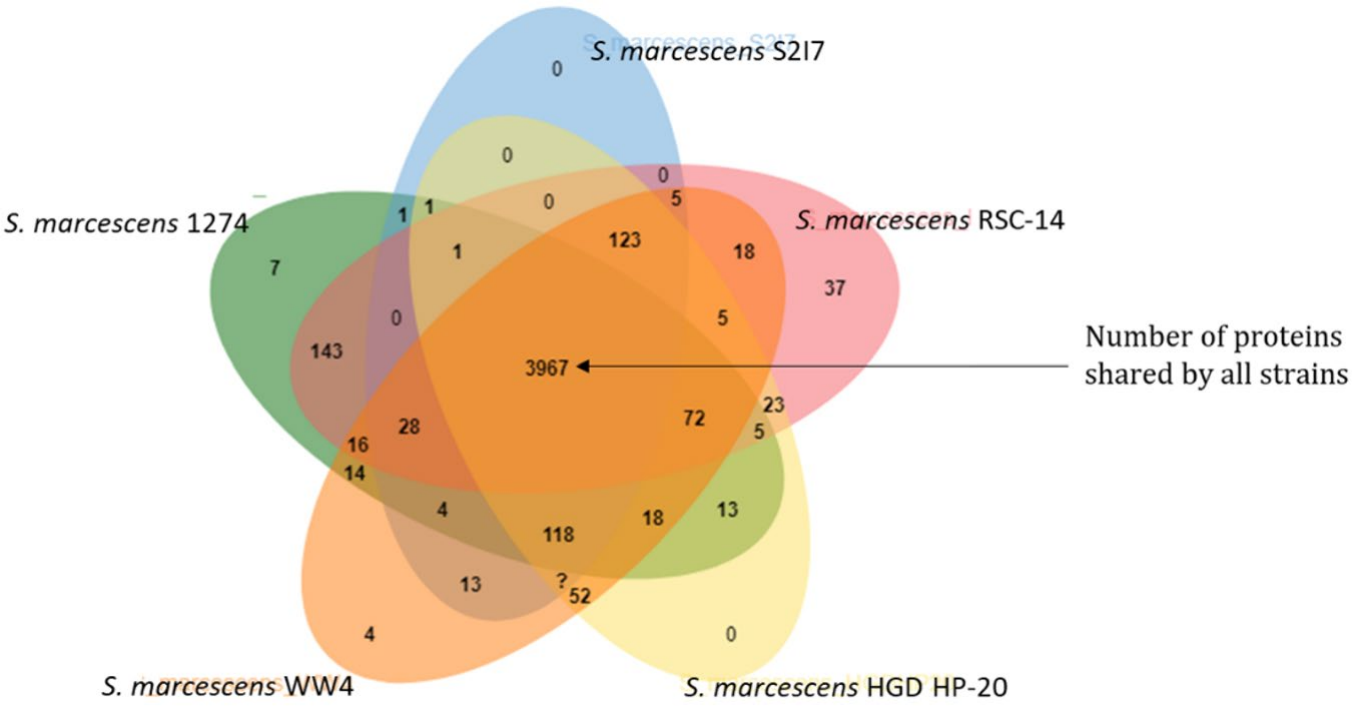
Cluster of proteins shared by the selected bacterial genomes.

The cluster of orthologous groups of proteins in the selected genomes was analyzed as annotated by IMG (Integrated Microbial Genomes) database^12^ (Table 4). The homology of the proteins in the selected genomes was analyzed using Orthovenn-2^13^. The selected strains formed 4883 clusters consisting of 938 orthologous clusters (at least contains two species) and 3945 single-copy gene clusters. The genomes shared 3967 proteins common to all (Fig. 9), while the query genome *S. marcescens* S2I7 contain 4485 proteins; 4456 proteins were present in clusters and 15 proteins were found as a singleton.

**Table 4:** COG classification of in *Serratia marcescens* strains S2I7, 1274, EGD HP20, RSC-14, and WW4. Clusters of orthologous gene groups were retrieved from the IMG (Integrated microbial genome and microbiome) database.

COG category	Class Id	S2I7		1274		EGD HP-20		RSC-14		WW4	
		CDS	%	CDS	%	CDS	%	CDS	%	CDS	%
Amino acid transport and metabolism	[E]	450	10.58	270	5.22	451	10.52	453	10.52	450	10.31
Carbohydrate transport and metabolism	[G]	376	8.84	230	4.45	375	8.74	373	8.66	378	8.66
Cell cycle control, cell division, chromosome partitioning	[D]	41	0.96	72	1.39	41	0.96	41	0.95	41	0.94
Cell motility	[N]	86	2.02	96	1.85	95	2.21	107	2.48	115	2.63
Cell wall/membrane/envelope biogenesis	[M]	253	5.95	188	3.64	256	5.97	264	6.13	254	5.82
Chromatin structure and dynamics	[B]	1	0.02	19	0.36	1	0.02	1	0.02	1	0.02
Coenzyme transport and metabolism	[H]	231	5.43	179	3.46	228	5.32	224	5.2	230	5.27
Defense mechanisms	[V]	95	2.23	46	0.89	97	2.26	89	2.07	102	2.34
Energy production and conversion	[C]	249	5.85	258	4.99	249	5.81	242	5.62	249	5.7
Extracellular structures	[W]	23	0.54	1	0.02	26	0.61	30	0.7	34	0.78
Function unknown	[S]	245	5.76	1347	26.08	244	5.69	242	5.62	256	5.86
General function prediction only	[R]	366	8.61	702	13.59	369	8.6	361	8.38	374	8.57
Inorganic ion transport and metabolism	[P]	280	6.58	212	4.1	281	6.55	290	6.73	283	6.48
Intracellular trafficking, secretion, and vesicular transport	[U]	67	1.58	158	3.06	67	1.56	73	1.7	68	1.56
Lipid transport and metabolism	[I]	154	3.62	94	1.82	156	3.64	152	3.53	157	3.6
Mobilome: prophages, transposons	[X]	10	0.24	—	—	23	0.54	19	0.44	31	0.71
Nucleotide transport and metabolism	[F]	106	2.49	95	1.84	106	2.47	111	2.58	106	2.43
Posttranslational modification, protein turnover, chaperones	[O]	164	3.86	203	3.93	164	3.82	168	3.9	168	3.85
RNA processing and modification	[A]	1	0.02	25	0.48	1	0.02	1	0.02	1	0.02
Replication, recombination and repair	[L]	111	2.61	238	4.6	116	2.7	112	2.6	115	2.63
Secondary metabolites biosynthesis, transport and catabolism	[Q]	110	2.59	88	1.7	112	2.61	119	2.76	113	2.59
Signal transduction mechanisms	[T]	185	4.35	152	2.94	190	4.43	192	4.46	183	4.28
Transcription	[K]	397	9.33	231	4.47	402	9.37	402	9.34	410	9.39
Translation, ribosomal structure and biogenesis	[J]	252	5.93	245	4.74	239	5.57	240	5.57	242	5.54
Not in COG		885	19.05	—	—	1059	21.89	1087	22.28	1066	21.72

### Validation of rhizodegradation of benzo(a)pyrene in the rhizosphere of *M. azedarach* plants under the stress of Cd

**Figure 10:**
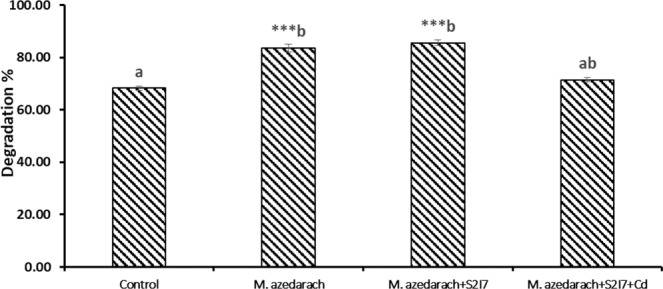
Rhizodegradation efficiency of the *M. azedarach* and *S. marcescens* association after 60 days. Statistical analysis, one-way ANOVA was done followed by post hoc analysis. Groups bearing the different superscript are significantly different from each other. Values are significantly different from control: *p < 0.05; **p < 0.01; ***p < 0.001.

The rhizodegradation of BaP was determined in the rhizosphere of *M. azedarach* after 60 days, analyzing the residual amount of BaP in the soil through GC-MS analysis (GCMS data and chromatogram in Supplementary Data). The initial concentration of BaP in the trials was 50 mg/kg and after 60 days the final concentration in the trial where the plant-microbe association (*M. azedarach* + *S. marcescens S2I7*) was applied was found to be 7.22 mg/kg. Therefore, the efficiency of degradation was 86%. However, in the presence of Cd, the efficiency was decreased by 15%. Another trial where no bacteria was augmented, the final concentration was found to be 8.29 mg/kg (degradation 83%). Most importantly, in the bulk soil without the plant-microbe interaction, the efficiency of degradation was significantly less (Fig. 10). The organic carbon content, N-content, P-content and pH of the soil has been monitored during the process of degradation. The percent of organic carbon content was found to be in the range of 0.75-1.5. The initial pH of the soil was 6.8 and after 60 days, the pH of the soil was found to be 6.9 (bulk soil), or close to neutral in other trials with plants.

## Discussion

The co-contamination of high molecular weight PAH, such as BaP, along with metals like Cd is the cause of concern and limits the strategies of bioremediation. Therefore, the process of rhizodegradation should start with the isolation of microbial strain with the capability to degrade PAH, resistance to other inorganic co-contaminants and capable to promote plant growth efficiently in stress conditions. Out of several bacterial isolates, *S. marcescens* S2I7 was found to be an excellent BaP degrader along with its ability of Cd resistance and due to its versatile characteristics, the genome of the isolate was sequenced and characterized. Earlier also, *Serratia* spp. have been reported as a plant growth stimulator in Cd contaminated soils. Khan *et al*. (2017) reported that *S. marcescens* RSC-14, an isolate from the roots of the Cd-hyperaccumulator *Solanum nigrum*, was highly resistant to Cd and could alleviate the heavy-metal stress in the host plant^9^. Similarly, Luo *et al*. (2011) reported an endophytic Cd resistant strain, *S. nematodiphila* LRE07, which promoted plant growth under Cd contamination^14^. These prodigiosin producing *S. marcescens* strains are considered as non-pathogenic, plant growth-promoting soil bacteria. The isolate S2I7 also has prodigiosin producing ability and versatile characters of resistance to Cd promoting the growth of plants and degrade BaP.

Bacterial species follow different rates, molecular mechanisms, and pathways for a breakdown of PAHs^15^ and different earlier studies have reported different bacterial species capable to degrade BaP. *B. subtilis* BMT4i could degrade more than 80% of BaP after 28 days of incubation^16^. *Cellulosimicrobium cellulans* CWS2, a novel strain degraded 78.8% of BaP after 13 days when the initial concentration applied was 10 mg/l^17^. Rentz *et al*. (2009) also reported the degradation of BaP by *Sphingomonas yanoikuyae* JAR02 when induced with salicylate and succinate and produced pyrene-8-hydroxy-7-carboxylic acid and pyrene-7-hydroxy-8-carboxylic acid as a by-product^18^. In the present study, 80.4% of BaP was degraded within 21 days by *S. marcescens* S2I7. In previous studies, effective degradation of BaP has been reported with the production of several metabolites, as analyzed by GCMS technique. Moody *et al*. (2004) identified several metabolites including trans-benzo[a]pyrene-11,12-dihydrodiol, cis-benzo[a]pyrene-11,12-dihydrodiol, cis-benzo[a]pyrene-4,5-dihydrodiol, 10-Oxabenzo[def]chrysene-9-one from BaP degradation pathway by *Mycobacterium vanbaaleni* PYR-1^19^. In the present study also, different metabolites including phthalate, salicylic acids were identified in the process of BaP degradation. For the degradation of high molecular weight PAHs, the bacterial species need to cope up with the abiotic stress. Therefore, the stress-responsive enzyme, GST plays a key role in Glutathione s-transferases (GSTs) in their survival under stress conditions. GSTs are part of a superfamily of enzymes that play a key role in cellular detoxification that is found in a wide range of living organisms and is associated with phase II detoxification of various environmental xenobiotic compounds^20^. Bacteria possess multiple GST genes of widely divergent sequences and unknown function^21^. The strain S2I7 also showed GST activity in the presence of Cd which was increasing with time^22^, which was later validated with the presence of GST genes in the genome. Conjugation of GSH with metal ions for cellular efflux is GST-dependent and is related to the stress response of cell^16^. In an interesting study, Simarani *et al*. (2016) reported that the GST activity of crude enzymes from rhizosphere isolate was different in comparison to purified GST enzyme^23^. Where the crude enzyme showed the activity of 5.78 × 10-06 µmol/min/mg using CDNB as a substrate in comparison to the specific activity of the purified enzyme of 0.264 ± 0.038 nmol/min/mg.

Different oxygenase (di- and mono-) enzymes and genes related to aromatic compound degradation have been reported from a variety of bacterial isolates in different studies. In the present study, the genome study of *S. marcescens* S2I7 revealed the presence of operons and genes of dioxygenase and mono-oxygenase families that are responsible for the catabolism of aromatic compounds. *S. marcescens* S2I7 produced both enzymes – i.e. catechol 1, 2 dioxygenase and catechol 2,3 dioxygenase, which was further validated by the presence of respective coding gene clusters in its genome as - Catechol 1, 2 dioxygenase (C12D) (929 bp; Position 3298937-3299866) and catechol 2, 3 dioxygenase (C23D) (812 bp; Position 659279-660091). Although, the C23D was produced in a higher amount as compared to C12D, by *S. marcescens* S2I7. In a previous report too, such variation has been noticed and attributed to the relatively lesser complex pathway for C23D, than C12D^24^. The economy of energy used by the bacteria for the production of these two enzymes, in the presence of PAH, has a major impact on their quantities.

More than 540 genomes of genus *Serratia* have been sequenced completely or draft and submitted to the NCBI database. However, to our knowledge, genome-wide researches regarding the bioremediation of organic compounds by *Serratia* strains are very rare, and here we describe systemic genome analysis revealing the potential of hydrocarbon degradation and metal resistance in isolate *S. marcescens* S2I7, which has not been described before. The phylogenomics analysis of the genome revealed the similarity distance with other sequenced genomes. Interestingly, the closely related strain EGD-HP20 was also reported from India that was reported to possess the excellent proteolytic activity and was utilized in the biodegradation of poultry waste^25^. The clinical isolates, such as NTCT13920 was found to be distantly related. The plant growth-promoting attributes of the strain *S. marcescens* S2I7, along with metal resistance and hydrocarbon degradation suggests its great potential for rhizoremediation of petroleum waste. The comparative analysis of the selected genomes revealed the relatedness among the isolates and their orthologous genes.

The strain S2I7 showed good PGP activities. The genetic basis of phosphate solubilization of bacteria is not fully understood. However, glucose dehydrogenase-pyrroloquinoline quinone is necessary for a primary mechanism of phosphate solubilization^26^. Glucose dehydrogenase (*gdh*) with pyrroloquinoline quinone (PQQ) as cofactor produces organic acids that are considered to be responsible for phosphate solubilization. The presence of a gene cluster containing the *gdh* gene and *pqq* genes confirmed the P-solubilizing ability of the isolate. Apart from that the presence of a gene cluster for IAA production and enterobactin type siderophore further established the PGP characteristics of the isolate. Bacteria have been reported to follow different tryptophan-dependent pathways for biosynthesis of IAA and form different intermediates such as indole-3-pyruvate (IPyA), indole-3-acetamide (IAM), indole-3-acetonitrile (IAN), tryptamine (TAM), and tryptophan side chain oxidase^27^. The presence of the Indole-3-acetylaspartic acid hydrolase (*iaaH)* gene in the position of 4510254–4511570 suggested that the strain S2I7 follow tryptophan-depended IAM pathway for IAA production.

The contamination of metals and PAHs in the soil leads to persistent accumulation in soil particles that have negative impacts on soil health^28^. Plants contribute to the increase in degradation of contaminants particularly in rhizospheric soil, due to increased microbial population and interaction^29^. Several earlier studies have reported a variety of plant species that enhanced the degradation of aromatic compounds in the rhizosphere. Chekol *et al*. (2004) reported different plant species like alfalfa, flat pea, sericea lespedeza, deer tongue, reed canarygrass, switchgrass, and tall fescue that could significantly reduce PCB concentrations in comparison to the unplanted controls^30^. Pradhan *et al*. (1998) also reported enhanced removal of PAH in the rhizosphere, when compared to un-planted controls^31^. However, still, studies are going on to find out more efficient plants to promote rhizodegradation. The rhizoremediation process utilizes the natural potential of plant-microbe association in the degradation of organic pollutants^32^. In the process of rhizodegradation, it is believed that bacteria or other microorganisms play a crucial role. In the rhizosphere, the plant provides exudates like sugars, amino acids, enzymes, and other compounds that stimulate bacterial growth and the additional surface area for microbes to grow. Thus the increase in the population of microorganisms and availability of contaminants in the rhizosphere than in non-rhizospheric soil do increase the interaction and efficiency in degradation^33^.

Plant’s ability to tolerate the contaminants and extensive root systems are two major factors for rhizoremediation^34^. Therefore, the selection of suitable plant species plays an important role in rhizoremediation. The plant of the present study *M. azedarach* is very common in the contaminated area and recently we have also reported efficient rhizodegradation of BaP in the rhizosphere of *M. azedarach* plant using surfactin producing bacilli strains^35^. Similarly, the strain S2I7 offers growth of plant *M. azedarach* leads to rhizoremediation of metal and PAH-contaminated soil. It was found that the application of strain S2I7 led to increasing in rhizodegradation of BaP, which was greater than in bulk soil. Moreover, the application of the isolate increased the degradation in comparison to the section where no efficient bacterial isolate was added. Other plants also have been reported to enhance rhizodegradation. Lu *et al*. (2011) also reported efficient degradation of PAHs (Phenanthrene and Pyrene) in the rhizosphere of mangrove *Kandelia candel* (L.) Druce after 60 days^36^. In another study, a higher efficiency (87%) in the remediation of total petroleum hydrocarbon was observed in the rhizosphere of *Rhizophora mangle* L. after 90 days with enhanced growth of bacteria in its rhizosphere^37^. Similarly, *Serratia marcescens* RSC-14 was reported to promote plant growth and phytoextraction of Cd with hyperaccumulator plant *Solanum nigrum*. However, unlike this report, *S. marcescens* RSC-14 was unable to degrade PAHs and lack siderophore production ability, essential for sequestration of iron^9^.

## Conclusion

The plant-microbe systems for bioremediation of PAH face challenges of co-contaminations of metals, and therefore require efficient symbionts for successful degradation. The use of *S. marcescens* S2I7 – *M. azedarach* pair for rhizoremediation of benzo(a)pyrene in Cd co-contaminated soil was found to be highly effective. The versatile characteristics of S2I7, which included its Cd- resistance and plant growth-promoting attributes, in addition to efficient PAH degradation ability, recommends it for the role of rhizoremediation. The analysis of *S. marcescens* S2I7 genome revealed its genetic background for these characteristics, and several other features such as physiological mechanisms available for stress response, phosphate solubilization, IAA and siderophore release, along with a tolerance for cadmium, arsenic and other metals. Conclusively, because of the wide array of attributes, and for being effective in PAH degradation, *S. marcescens* S2I7 may be highly useful with *M. azedarach* rhizosphere for environmental applications in PAH and metal co-contaminated soils.

## Materials and method

### Isolation and screening of BaP degrading bacterial strains

The bacterial strains were isolated via selective enrichment where contaminated soil sample was inoculated into the mineral salt medium (MSM) amended with benzo(a)pyrene (2 mM) as reported earlier^38^. Enrichment was conducted at 30 °C and 120 RPM on a rotary shaker, incubated for about 7 days and was carried out in three consecutive batches, each for 7 days. The growth of the enriched cultures was monitored by measuring the turbidity at 600 nm (600_OD_). Further, the isolated bacterial strains were screened for degradation of BaP by growing on minimal media agar plates amended with BaP.

The preliminary screening for the degradation of BaP was quantified with a colorimetric assay. For that, the isolated bacterial strains were inoculated into Bushnell-Haas broth amended with BaP (2 mM) and Methylene blue (2% v/v as redox indicator), and incubated at 30 °C with constant shaking at 180 rev/min, for 14 days. The set without bacterial inoculation was used as control. From broth culture, a 5 ml sample was centrifuged at 6000 rev/min for five minutes and the supernatant was assayed spectrophotometrically at 609 nm for the residual hydrocarbon, and PAH degradation percentage was determined using the following equation^39^.

Percentage of PAH degradation = 1 − (Absorbance of sample)/(Absorbance of Control) × 100

### Determination of cadmium resistance of the isolates

The isolated BaP degrading bacterial isolates were checked for their resistance to cadmium (Cd). The isolated strains were grown in Mueller Hinton Agar with 0.25 mM of cadmium (CdSO_4_) at 30 °C to screen the Cd-resistance. The positive isolates were further analyzed for their maximum tolerable concentration (MTC) of Cd with increasing concentration of Cd. The starting concentration of Cd was 0.25 mM and the culture were grown on medium with subsequent higher concentration (0.5 mM, 0.75 mM, 1 mM, 1.25 mM, 1.5 mM, 1.75 mM, 2 mM, 2.25 mM, 2.5 mM, 2.75 mM, 3 mM, 3.25 mM, 3.5 mM, 3.75 mM, 4 mM, 4.25 mM, 4.5 mM, 4.75 mM, 5 mM) of CdSO_4_.

### Determination of plant growth-promoting attributes of the isolates

PGP attributes like phosphate solubilization, IAA production, HCN production, and siderophore production were tested according to the protocols described in our earlier reports^38^. To determine the Phosphate solubilization activity, the isolates were grown in Pikovaskay’s medium with tricalcium phosphate as insoluble phosphate and amended with bromophenol blue. The formation of a clear yellow color halo around the colonies was considered as Positive phosphate solubilization. To check the production of IAA, the isolates were grown in tryptone-yeast medium and incubated in dark on an orbital shaker at 200 RPM for 72 hours. One ml of culture supernatant was mixed with 1 ml of Salkowsky’s reagent and incubated in dark for 30 minutes and measured spectrophotometrically at 536 nm and quantified using the standard.

Siderophore production ability of the isolates was determined on solid CAS (Chrome Azurol S) medium and the production of siderophore was confirmed with the formation of the orange/yellow circle around the colonies after incubating at 30 °C for 7 days. To determine the production of hydrogen cyanide (HCN), the isolates were grown on nutrient agar medium supplemented with glycine (4.4 g/L) and the inner side of the lid was covered with a Whatman filter paper pre-soaked in a specific solution (0.5% picric acid and 2% sodium carbonate w/v). Plates were incubated at 37 °C for 4 days, sealed with Parafilm paper and the appearance of an orange or red color indicates the production of hydrogen cyanide^40^.

### Phylogenetic analysis of 16S rDNA

Molecular phylogenetic analysis was done for the 16 s rDNA sequence of the selected strain. 16 S rRNA genes were sequenced after amplification by polymerase chain reaction (PCR) using primers 27F-5ʹ-AGAGTTTGATCMTGGCTCAG-3ʹ and 1492R-5ʹ-TACGGYTACCTTGTTACGACTT-3ʹ according to conditions described earlier^34^. 16 S rDNA sequence was compared with other sequences in Gen Bank using (http://www.ncbi.nlm.nih.gov) and aligned. The sequences were submitted in NCBI.

### Estimation of Catechol 1,2 dioxygenase and Catechol 2,3 dioxygenase activity of the isolate

The activity of Catechol 1,2 dioxygenase (C12D) and Catechol 2,3 dioxygenase (C23D) of the selected isolate were assayed spectrophotometrically by measuring the rate of production of metabolites from catechol. C12D activity was measured at 260 nm by observing the formation of cis,cis-muconic acid and the activity of C23D was measured at 375 nm by determining the formation of 2-hydroxy muconic semi-aldehyde from catechol after every 24 hours of incubation up to 120 hours^41^.

### Quantitative analysis of the degradation of BaP and effects of Cd and other carbon sources (Succinate) on it

The degradation of BaP and the formation of metabolites by the bacterial isolate was analyzed and confirmed by GC-MS analysis. The bacterial strain was grown in BaP (2 mM) amended Bushnell Haas broth and the aliquots from the flask were extracted with three equal volumes of ethyl acetate after every 7 days for 21 days. The effects of Cd and other carbon source was determined by adding 0.25 mM CdSO4 and sodium succinate (10 mg/l) in the medium. After initial extraction, the aqueous fraction was acidified with concentrated HCl to pH 2 and again extracted equal volumes of ethyl acetate.

### Genome sequencing, assembly, and annotation

The genome of the selected isolate was sequenced with whole-genome shotgun sequencing and was done using one Illumina paired-end library with an average insert size of ~400 bp. Illumine Truseq Nano DNA Library Prep. the kit was used to prepare the paired-end sequencing libraries after fragmented by Covaris M220 that generates dsDNA fragments with 3′ or 5′ overhang. The fragments were then subjected to end-repair followed by adapter ligation to the fragments. The products were then PCR amplified with the index primer as described in the kit protocol and sequenced using Next Seq500. The paired-end reads generated using the NextSeq500 generating ~1 Gb of raw reads^42^.

After sequencing, the raw data was processed to obtain high-quality clean reads using Trimmomatic v0.35 to remove adapter sequences, ambiguous reads, and low-quality sequences. These reads were trimmed using a quality score threshold of 20 and a length cutoff of 20 bp. Reference-guided assembly of the sample was performed using Samtools^43^. The procedure for genome annotation was done by the RAST (Rapid Annotation using Subsystem Technology) server and NCBI prokaryotic genome annotation pipeline (https://www.ncbi.nlm.nih.gov/genome/annotation)^44,45^. The rRNA and tRNA genes were predicted and annotated using RNAmmer^46^ and tRNAscan-SE^47^ respectively.

The sequenced genome was further analyzed and annotated through the Bacterial Annotation system (Basys)^48^ (https://www.basys.ca), RAST annotation server (http://rast.theseed.org/FIG/rast.cgi)^44^ and dfast annotation. The target genes, operon and their position in the genome were analyzed and compared.

### Comparative genomic analysis

The genome of the strain S2I7 was compared based on its sequence with other *Serratia marcescens* genome present in the NCBI database (https://www.ncbi.nlm.nih.gov/genome/?term=serratia+marcescens) (Retrieved on January 2020). Then the genome was compared with its most closely related strain (*Serratia marcescens* EGD HP20) and one of the most distantly related strains (*Serratia marcescens* NCTC13920) based on its nucleotide sequence using the Artemis Comparison Tool (ACT)^49^. Apart from that, the genome of S2I7 was compared based on its annotated features with four other soil bacterial isolates of the same species group, *S. marcescens* 1274, *S. marcescens* RSC-14, *S. marcescens* WW4 and *S. marcescens* EGD HP20 using Mauve 2.0^50^. The annotated data of the genomes were retrieved from the IMG JGI database^12^, and the Cluster of orthologous groups (COG) functional annotation were done in WebMGA. The annotated features and protein-coding genes were clustered and compared using orthovenn 2.0^13^.

### Validation of rhizodegradation potential of the isolate

A pot experiment was done to determine the effects of inoculation of *S. marcescens* with *Melia azedarach* in the rhizodegradation of BaP and also the effects of Cd co-contamination on degradation. The *M. azedarach* seeds were washed with water and surface sterilized with 0.1% HgCl_2_ for 2–3 min followed by washing with ethanol and distilled water. The seeds were soaked in sterile water and kept in a rotary shaker for 48 h. and then were grown in sterile soil for 20 days. Then the seedlings of equal size were transferred to pots containing non-sterile soil (C), Soil + PAH (P), or SoilvPAH + Isolate S2I7 (P27), Soil + PAH + Cd + S2I7 (PC27) and grown for 60 days. The isolates were inoculated on to the roots of seedlings by dipping it in 10 ml (O.D. 600 = 0.5) of bacterial suspension of late log phase, in sterile conditions, before transferring in the soil of respective treatment^51^.

At the end of the pot trial experiment, 1 gm of soil was collected and suspended in 25 ml of acetonitrile (ACN) and extracted for 15 min by ultrasonic stirring and then centrifuged at 10000 RPM for 12 mins. Aliquots of 1 ml of each sample were diluted in 10 ml ACN and analyzed using GC-MS. EPA (Environmental Protection Agency) 610 mix was used as certified reference material for standardization. Benzo[e]pyrene-d12 perdeuterated was used as an internal standard. The analytical process was also validated by using pure BaP at different concentrations for preparing the calibration curve.

The degradation of BaP in the soil for the treatments after 60 days of cultivation was calculated by the following equation^52^.

$${\rm{BaP}}\,{\rm{degradation}}\,{\rm{in}}\,{\rm{soil}}\,( \% )=\frac{(Co-C)}{Co}\times 100$$

where Co is the initial concentration of BaP.

C is the concentration of BaP in the soil after 60 days.

## Supplementary information

Supplementary information
