# Supplementary material for: Rhizosphere assisted biodegradation of benzo(a)pyrene by cadmium resistant plant-probiotic Serratia marcescens S2I7, and its genomic traits
Source: Sci Rep. 2020 Mar 24;10:5279. doi: 10.1038/s41598-020-62285-4 (PMC7093395; doi:10.1038/s41598-020-62285-4)

Title: Rhizosphere assisted biodegradation of benzo(a)pyrene by cadmium resistant plant-probiotic *Serratia marcescens* S2I7, and its genomic traits.

Authors: **Rhitu Kotoky**
Department of Microbiology,
Assam University, Silchar
Assam, India-788011.

Piyush Pandey
Department of Microbiology,
Assam University, Silchar
Assam, India-788011

Corresponding author: Piyush Pandey

Email address: piyushddn@gmail.com

Supplementary file 1:

Maximum tolerable concentration of Cd showed by the bacterial isolates.

**Isolates**	Maximum tolerable concentration (MTC) (mM)
**S1I1**	0.75
**S1I5**	1
**S1I7**	1
**S1I8**	1
**S1I2**	0.5
**PDB_4**	0.75
**S2I3**	2.75
**SR1**	1.5
**S2I5**	3
**S2I7**	3.25
**S1I21**	1.25
**S1I24**	1
**S1I26**	1
**S3I12**	1.25

Supplementary file 2:

Colorimetric assay of degradation BaP by the isolates.

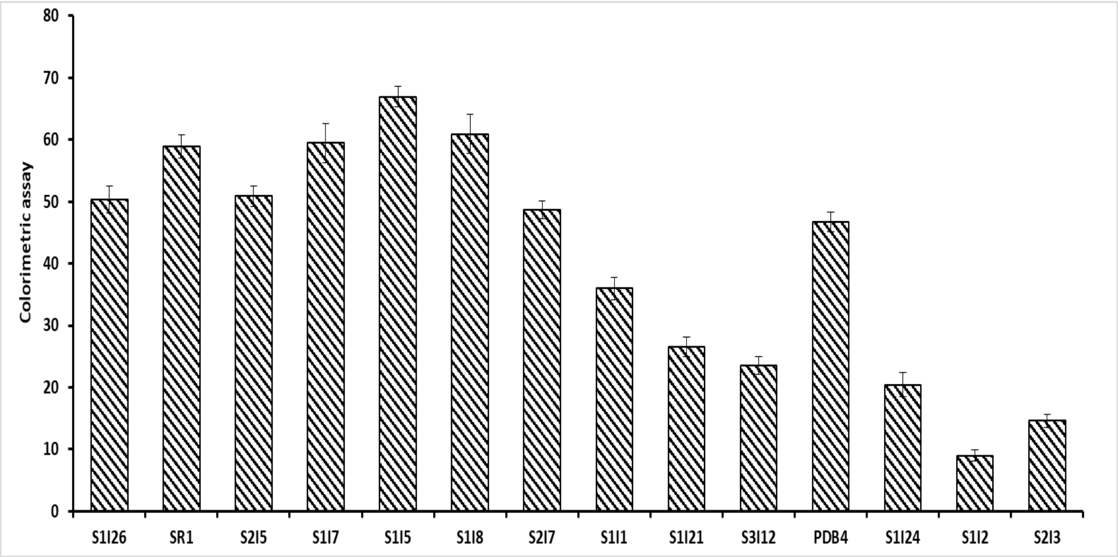

<u>Supplementary file 3:</u>

Plant growth promoting (PGP) attributes of the isolates. Where P-positive and N-negative.

Isolates	PGP attributes			
	P-Solubilization	Siderophore production	IAA production (µg/ml)	HCN production
S1I1	N	P	9.1±0.2	P
S1I5	P	N	7.9±0.35	N
S1I7	P	N	7.6±0.2	N
S1I8	P	P	11.5±0.56	P
S1I2	N	N	8.9±0.35	P
PDB_4	P	N	8±0.2	P
S2I3	N	N	12.9±0.78	N
SR1	P	N	11.1±0.9	P
S2I5	N	N	14.2±0.9	P
S2I7	P	P	49.8±2.01	P
S1I21	N	N	8.1±0.45	P
S1I24	N	N	8.4±0.5	N
S1I26	P	P	8.1±0.55	N
S3I12	N	N	8.7±0.8	N

Supplementary file 4:

Venn analysis of 14 isolates capable to degrade BaP on the basis of their PGP activity and Cd resistance. For the analysis the isolates with resistance for Cd concentration more than 2.75M along with the isolates producing IAA more than 20 µg/ml were selected.

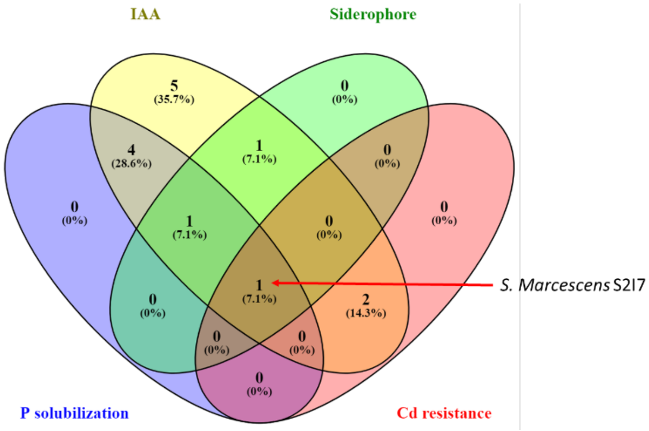

Supplementary file 5:

Catechol 1, 2 dioxygenase and Catechol 2,3 dioxygenase activity of *S. marcescens* S2I7.

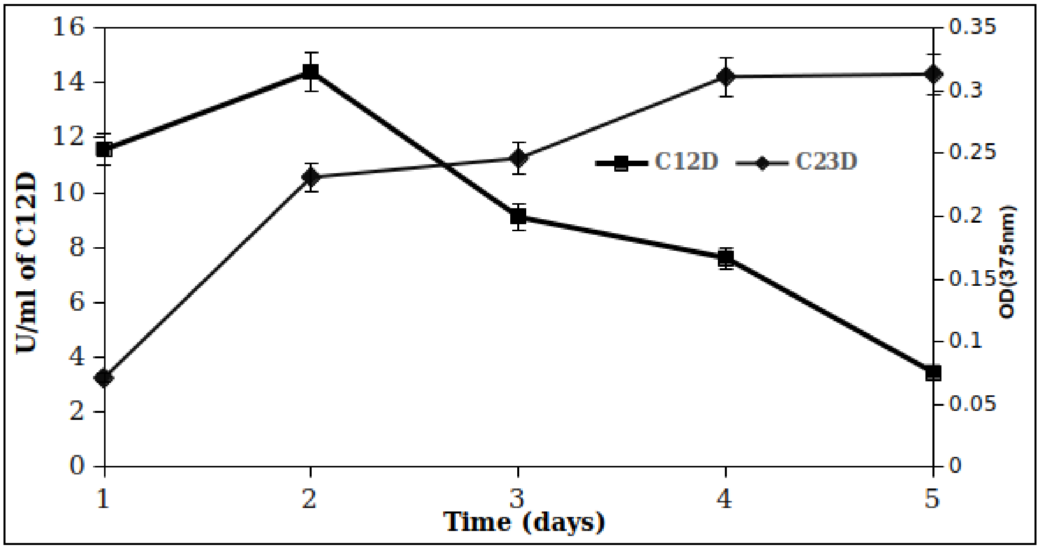

Supplementray file 6:

GC-MS chromatograms of degradation of BaP by *S. marcescens* S2I7, which were analyzed after day 7 (A), after day 14 (B) and after day 21 (C). The abundance (%) has been shown by Y-axis, where the peak for BaP revealed at retention time-33.94 mins.

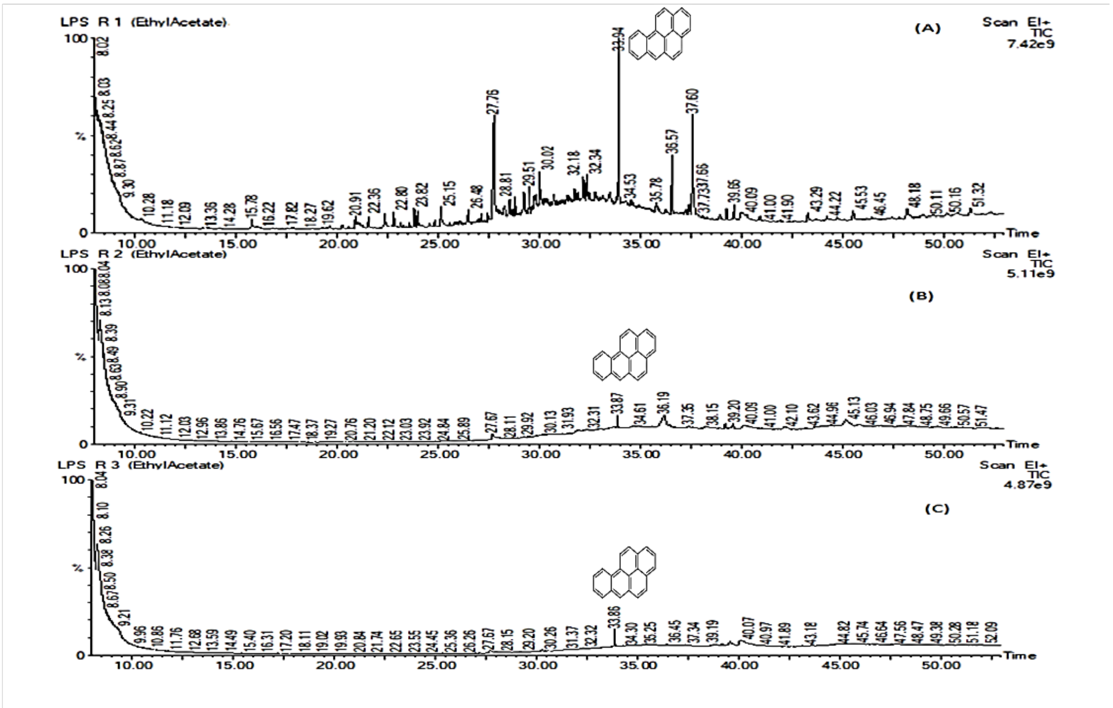

Supplement: Supplementary file 1 — Supplementary information [file 41598_2020_62285_MOESM1_ESM.pdf]
